# Vibration paradox in orthodontics: Anabolic and catabolic effects

**DOI:** 10.1371/journal.pone.0196540

**Published:** 2018-05-07

**Authors:** Mani Alikhani, Sarah Alansari, Mohammad A. Hamidaddin, Chinapa Sangsuwon, Bandar Alyami, Soumya N. Thirumoorthy, Serafim M. Oliveira, Jeanne M. Nervina, Cristina C. Teixeira

**Affiliations:** 1 Advanced Graduate Education Program in Orthodontics, Department of Developmental Biology, Harvard School of Dental Medicine, Boston, Massachusetts, United States of America; 2 The Forsyth Institute, Cambridge, Massachusetts, United States of America; 3 Consortium for Translational Orthodontic Research, Hoboken, New Jersey, United States of America; 4 Department of Orthodontics, New York University College of Dentistry, New York, New York, United States of America; 5 Department of Mechanical Engineering, Polytechnic Institute of Viseu, Portugal; 6 Department of Basic Science & Craniofacial Biology, New York University College of Dentistry, New York, New York, United States of America; Charles P. Darby Children's Research Institute, 173 Ashley Avenue, Charleston, SC 29425, USA, UNITED STATES

## Abstract

Vibration in the form of High Frequency Acceleration (HFA) is anabolic on the craniofacial skeleton in the absence of inflammation. Orthodontic forces trigger an inflammation-dependent catabolic cascade that is crucial for tooth movement. It is unknown what effect HFA has on alveolar bone if applied during orthodontic treatment. The objectives of this study are to examine the effect of HFA on the rate of tooth movement and alveolar bone, and determine the mechanism by which HFA affects tooth movement. Adult Sprague Dawley rats were divided to control, orthodontic force alone (OTM), and different experimental groups that received the same orthodontic forces and different HFA regimens. Orthodontic tooth movement was assessed when HFA parameters, frequency, acceleration, duration of exposure, and direct or indirect application were varied. We found that HFA treatment significantly enhanced the inflammation-dependent catabolic cascade during orthodontic tooth movement. HFA treatment increased inflammatory mediators and osteoclastogenesis, and decreased alveolar bone density during orthodontic tooth movement. Each of the HFA variables produced significant changes in the rate of tooth movement and the effect was PDL-dependent. This is the first report that HFA enhances inflammation-dependent catabolic cascades in bone. The clinical implications of our study are highly significant, as HFA can be utilized to enhance the rate of orthodontic tooth movement during the catabolic phase of treatment and subsequently be utilized to enhance retention during the anabolic remodeling phase after orthodontic forces are removed.

## Introduction

Orthodontic tooth movement depends on the rate of bone resorption, which in turn depends on osteoclast activity. Interestingly, osteoclasts are not resident cells in the periodontal ligament (PDL) or alveolar bone. Instead, orthodontic force stimulates release of inflammatory cytokines and chemokines in the periodontium, which recruits osteoclast precursors cells that undergo differentiation through signaling pathways–especially RANK-RANKL activation–resulting in a localized region of catabolic activity (bone loss) and subsequent tooth movement [1–8].

Interestingly, there is a saturation point in this biological response above which increasing the magnitude of the orthodontic force does not increase the level of inflammatory markers. It is the saturation point that accounts for the plateau in the rate of tooth movement even in the presence of high magnitude forces [9]. While this limitation exists for continuous and static orthodontic forces, it is not clear if any other form of mechanical stimulation, such as intermittent forces, can be used to bypass the saturation point.

Applying vibration in the form of high frequency acceleration (HFA) in the absence of any pathology or inflammation has an osteogenic effect on long bones and craniofacial bones, specifically alveolar bone [10, 11]. Likewise, applying HFA to healing bones, after the source of pathology has been eliminated, prevents extensive bone resorption and stimulates bone formation [12–14]. In both of these situations, HFA applied directly to the target site or indirectly to an adjacent site, does not induce any inflammatory mediators nor increase the number of osteoclasts.

Several prospective randomized controlled clinical trials, pilot studies, or animal studies have recently investigated the effect of vibration on orthodontic tooth movement [15–19]. The differences in these treatment outcomes are controversial and still unclear as some of these studies reported an increase in the rate of tooth movement, while others did not. Moreover, these studies have not identified isolated effects of vibration characteristics such as different acceleration, resultant load, frequency and duration of applied load, rendering interpretation of data very difficult. Furthermore, the underlying cellular and molecular basis of this phenomenon remains unclear.

Given the strong anabolic effect of HFA on healthy and healing bones where inflammatory mediators are low, one would hypothesize that HFA application would inhibit any process that is inflammation-dependent, such as orthodontic tooth movement. However, this hypothesis must be evaluated carefully. It is critical to recognize that conditions under which HFA is anabolic (i.e., when inflammatory mediators are at baseline or slightly above baseline and declining) are significantly different from conditions under which orthodontic tooth movement occurs (i.e., when inflammatory mediators are significantly elevated). Thus, it is unknown if HFA treatment remains anabolic when inflammatory mediators are elevated. In fact, one could justify the opposite hypothesis–that HFA treatment is catabolic when inflammatory mediators are elevated–based on data showing that mechanical force delays healing by exacerbating inflammation and promoting bone resorption [20–22]. Therefore, here we propose that applying HFA, which produces intermittent forces during orthodontic tooth movement, will enhance inflammation in the PDL and alveolar bone, increase the rate of bone resorption and accelerate the rate of tooth movement.

Given the uncertainty of how vibration impacts orthodontic tooth movement, we investigated whether HFA’s different components have an anabolic or potential catabolic effect on bone during orthodontic treatment. More specifically, the purpose of our study was to: 1) examine the effect of HFA on the rate of orthodontic tooth movement, 2) examine bone density changes following HFA treatment and 3) identify the mechanism by which HFA affects orthodontic tooth movement.

## Materials and methods

### Animals and study protocol

Sprague Dawley rats (206 adult males; average weight of 400 g, 120 days of age) were divided into Baseline, Control, Orthodontic Tooth Movement only (OTM), and multiple Experimental groups (see below) that received orthodontic forces and different mechanical stimulation regimens. The protocol was approved by New York University Institutional Animal Care and Use Committee (Protocol Number: 120203). Animals in OTM and Experimental groups received an orthodontic force on the maxillary right first molar using Sentalloy closing coils that were custom-made by GAC International (Bohemia, NY) to deliver 10cN or 25cN of force for 1 mm activation [2]. The Baseline group did not receive spring nor HFA, and Control group received spring without activation. Our previous study has shown that 10cN is close to saturation point while 25 cN is above saturation point [9].Both these forces are commonly used in rat models of orthodontic tooth movement [23]. All closing springs were calibrated as previously described [2]. Briefly, force on the activated springs was measured with a digital force gauge (Phase II Plus, Upper Saddle River, NJ) at 37°C. Following inhalation anesthesia (3% isofluorane), springs were checked daily during the experimental period. Six animals with loose springs or springs that did not produce proper force were excluded.

**Fig 1:**
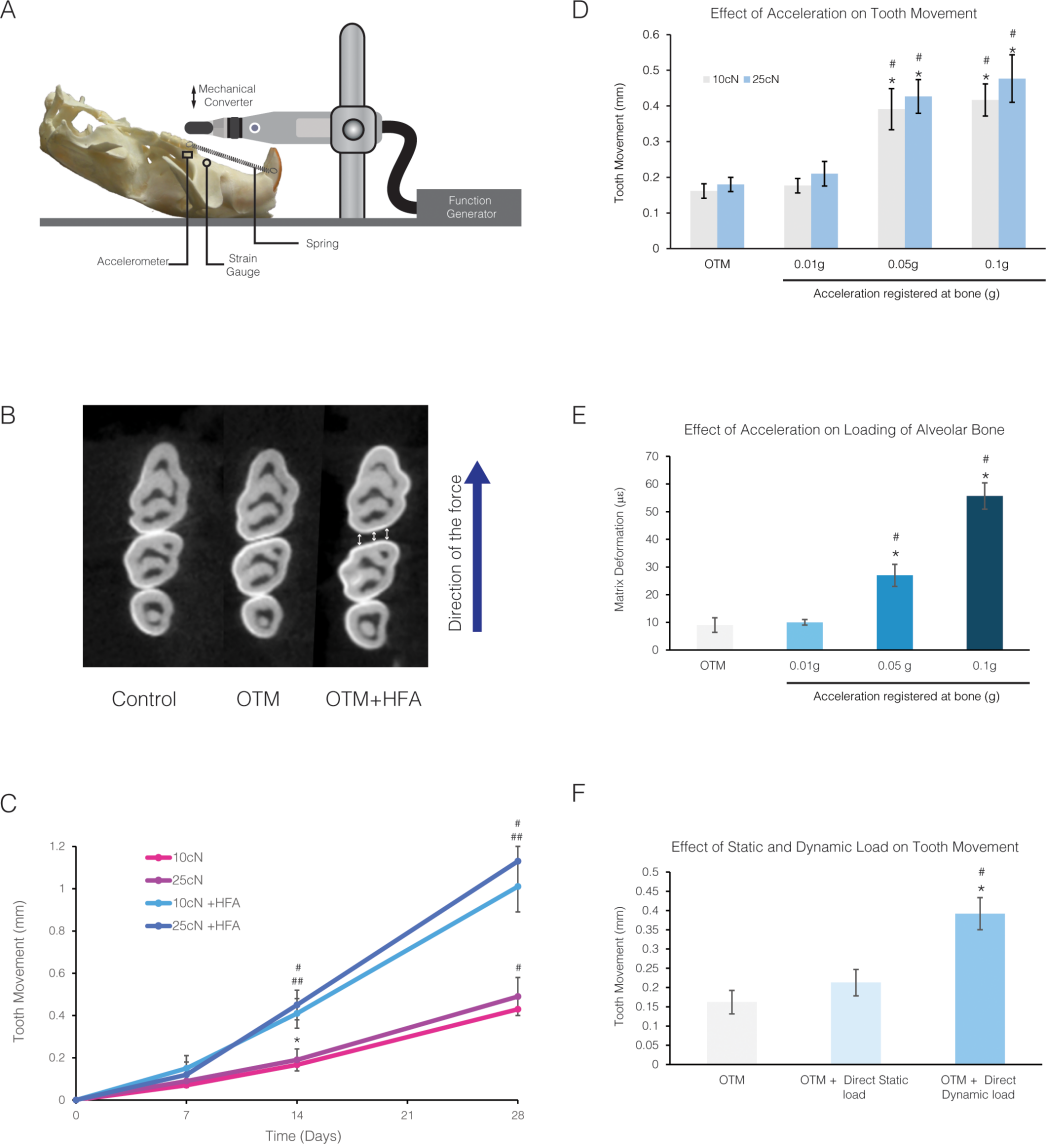
HFA effect on the rate of tooth movement depends on acceleration and the dynamic nature of the load. **(A)** Animals were exposed to orthodontic force alone or in combination with direct application of different HFA on the occlusal surface of maxillary right first molar using a device depicted in this schematic. Strain gauge and accelerometer are shown placed in the maxillary alveolar bone in the proximity of the first molar. **(B)** Tooth movement was measured in the axial slice from the microCT scan of maxillary right molars at the level maximum convexity (height of contour) for Control (no movement), OTM and OTM + HFA shown here for 14 days treatment with 10 cN force and 120Hz, 0.05g for 5 min a day. **(C)** Rate of tooth movement on day 7, 14 and 28 after application of 10cN or 25cN orthodontic forces in absence or presence of HFA for 5 min a day. Each value represents the mean ± SEM of 4 samples. (* Significantly different from day 0, for both 10 and 25 cN, # significantly different from all previous time points, for both for both 10 and 25 cN, ## significantly different from corresponding force group in the absence of HFA, for both 10 and 25 cN) **(D)** Effect of different accelerations on the rate of tooth movement at 120 Hz, in animals that received 10 cN or 25 cN orthodontic forces (* Significantly different from OTM group; ^#^ significantly different from 0.01g acceleration) **(E)** Average peak strain (mean ± SEM) in the alveolar bone surrounding the maxillary first molar in response to different accelerations with a set frequency of 120 Hz under 10 με load. HFA was directly applied to the occlusal surface of fresh rat skulls (n = 4) and average peak strain was registered using a strain gauge attached to the buccal and palatal surface of the alveolar bone close to upper right first molar (* Significantly different from OTM; ^#^ significantly different from 0.01g acceleration) **(F)** Effect of static or dynamic (120 Hz for 5 minutes per day for 14 days) load application to the first molar at a peak strain of 30 με (registered at the level of alveolar bone) on the rate of tooth movement, in the presence of 10cN orthodontic force (* Significantly different from OTM group; ^#^ significantly different from Static load group).

Experimental groups were exposed to different acceleration (0.01g, 0.05g, 0.1g), frequency (30Hz, 60Hz, 120Hz), and duration (5 or 10 minutes) protocols. Rats were anesthetized (3% isofluorane inhalation) and mechanical stimulation was applied (Fig 1A). Control and OTM groups did not receive vibration except for some Control group animals that were exposed to vibration as reported in the Results.

After force application, animals were euthanized by CO_2_ narcosis at Days 1, 3, 7, 14, and 28 and hemi-maxillae were collected. Four animals per group were used for tooth movement, micro-CT and histology studies, and 5 animals per time point were used for RNA and protein studies. Procedures were performed on one side of the maxilla, allowing the contralateral side to be used as an internal control.

### Anti-inflammatory treatment

Since inflammatory mediators are critical for orthodontic tooth movement and HFA is anabolic when inflammatory mediators are low, we examined the effects of non-steroidal anti-inflammatory drugs (NSAIDs) on HFA treatment during OTM. In these experiments, animals were exposed to similar orthodontic forces and HFA treatment as described above, and were given a daily dose of NSAID (Diclofinac 5mg/kg) administered intramuscularly (IM), changing injection sites to prevent discomfort to the animals. Animals were weighed daily to accurately calculate the dose of the medication for each animal and to ensure the rats were thriving.

### Acceleration and strain measurements

The vibration device was prepared and calibrated to deliver 0.01g, 0.05g or 0.1g gravitational acceleration at different frequencies (Mechanical Engineering Department of the Polytechnic Institute of Viseu-Portugal). Appliance calibration was performed with a sensor (OMRON-E2E-X7D1-N 23304; OMRON Electronics Iberia SAU, Lisbon, Portugal) that was connected to an oscilloscope (Metrix OX 803B 40 MHz, Metrix Electronics, Hampshire, United Kingdom) and a Digital Tachometer (Lutron DT 2236, Lutron Electronic Enterprise, Taipei, Taiwan). Strain gauges (UFLK-1-11-1L, 1 mm gauge length, 120 Ω TML Gages, Texas Measurements, College Station, TX, USA) were attached using cyanoacrylate to the palatal and buccal sides of the alveolar bone near the maxillary first molar on fresh and dry rat skulls. Strain signals were amplified by a low-noise amplifier (SX500, Beacon Dynamics, Byram Township, NJ, USA). Data acquisition and analyses were performed with the SPIDER 8 system and Catman 4.5 software measured with a piezoelectric sensor-Bimorph vibration element 4V5mm (Allied Electronics, Fort Worth, TX, USA), and a MotionNode 3-DOF inertial measurement unit (GLI Interactive, Seattle, WA, USA). Vibration was applied under very low static load of 10 με (microstrain), measured at the buccal alveolar bone surrounding upper first molar.

### Micro-CT imaging

Hemi-maxillae were scanned with a Scanco MicroCT (μCT40, Scanco Medical, Bassersdorf, Switzerland). Results were analyzed utilizing μCT V6.0 software on the HP open platform (OpenVMS Alpha Version 1.3–1 session manager). On occlusal sections, three reference points (buccal, middle, and palatal) were identified on the distal surface of the maxillary first molar and mesial surface of the maxillary second molar at the height of contour. The average distance between those points was calculated to quantify tooth movement. The Dahlberg [24] formula was applied to estimate the random errors and the paired t-test was applied to identify systematic errors according to Houston [25]. Random error for intra-observer evaluation was 0.015 mm and 0.023 mm for the inter-observer evaluation, and not statistically significant. Systematic errors were also small and not statistically significant (p = 0.886 for intra-observer and p = 0.852 for inter-observer). The area extending from the alveolar crest to 1 mm below the apex of the maxillary first molar was analyzed for bony changes (excluding the roots). This area is limited laterally by the buccal and lingual alveolar plates, and extended from the area between first and second molar roots to the area 0.5 mm mesial to the root of first molar. The ratio of bone volume to total volume (BV/TV) was calculated based on a threshold of 275.

### Histology and immunohistochemistry

Hemi-maxillae were fixed in 4% paraformaldehyde, demineralized in 14% EDTA solution for 2 weeks, dehydrated in alcohol series, embedded in paraffin, and cut into 5 μm sagittal sections. Five sections were stained with hematoxylin and eosin (H&E) and scanned on a Scan Scope GL series optical microscope (Aperio, Bristol, UK) at 20x magnification. Intermediate sections were immunostained with Cathepsin K antibody (Millipore, Billerica, MA) using Vectastain ABC kit (Vector Laboratories, Burlingame, CA). As a negative control, sections were exposed to pre-immune serum. Osteoclasts were defined as Cathepsin K–positive multinuclear cells on periosteal or endosteal bone surfaces along the full length of the mesial half of the mesiopalatal root. Osteoclasts were counted in five sections and values were averaged for each rat. Data were expressed as the mean number of Cathepsin K–positive cells per 1 mm^2^ area of PDL and adjacent alveolar bone. Two examiners completed all histological quantifications.

### Real-time PCR analysis

#### Total RNA extraction

Five animals from each group were sacrificed by CO_2_ narcosis at 24 hours, and the hemi-maxillae were dissected and frozen in liquid nitrogen. Total RNA was collected as described previously [26]

#### Real-time PCR

Inflammatory cytokines and cytokine receptor genes were analyzed with primers specific for rat genes, with a QuantiTect SYBR Green RT-PCR kit (both Qiagen, Valencia, CA) on a DNA Engine Optican 2 System (MJ Research, Waltham, MA). An mRNA pool for each group was tested three times. Relative levels of mRNA were calculated and normalized to the level of GAPDH and acidic ribosomal protein mRNA.

### ELISA analysis

Activity of inflammatory markers was measured by enzyme-linked immunosorbent assay (ELISA). Five hemi-maxillae from each group at each time point were dissected and frozen in liquid nitrogen and stored at -80°C. When ready for analysis, frozen hemi-maxillae were pulverized, lysates were prepared, and total protein quantitated using a BCA protein assay kit (Pierce, Rockford, IL). Concentration of interleukin (IL)-1ß (Thermo, Rockford, IL), tumor necrosis factor alpha (TNF-*α*) (Thermo), CCL2 (Abcam, Cambridge, MA), and RANKL (MyBioSource, San Diego, CA) were determined using ELISA. Data were analyzed in comparison to standard curves specific to each inflammatory marker.

### Statistical analysis

After confirming normal distribution of samples by the Shapiro-Wilk test, group comparisons were assessed by analysis of variance (ANOVA). Pairwise multiple comparison analysis was performed with the Tukey’s *post hoc* test. Two-tailed *p* values were calculated; *p* < 0.05 was set as the level of statistical significance.

## Results

### Increase in acceleration increased the rat of tooth movement

To characterize the effect of different components of vibration on the rate of tooth movement, we first studied the effect of vibration in the form of HFA (0.05 g, 120 Hz, 5 minutes) on the rate of tooth movement in the presence of 10 cN or 25 cN orthodontic force for 7, 14 and 28 days. The OTM group did not receive HFA treatment. Tooth movement was measured on axial micro-CT images at the level of the height of contour of the first and second molars (Fig 1B).

In comparison with day 0, the magnitude of tooth movement at days 7, 14 and 28 for both 10 cN and 25 cN was significantly increased (p<0.05) (Fig 1C). However, no significant difference in the rate of tooth movement between 10 cN and 25 cN was observed at any time point. In comparison with OTM group, application of vibration at day 14 significantly increase the rate of tooth movement 2.4-fold (10 cN group) and 2.5-fold (25 cN group) (p<0.05). Similarly, at day 28 days vibration caused a 2.3- and 2.4- fold increase in rate of tooth movement in 10 cN and 25 cN groups respectively, in comparison to the OTM group (p<0.05). Considering the change in the rate of tooth movement was similar after 14 and 28 days of force application, in the following results we present only 14 day data.

To evaluate the effect of acceleration on rate of tooth movement, different accelerations (0, 0.01, 0.05 and 0.1 g) were applied to the maxillary first molar, 5 minutes per day for 14 days at a constant frequency of 120 Hz. Each group received 10 cN or 25 cN orthodontic force during the experiment (Fig 1D).

The application of 0.01g acceleration did not increase the rate of tooth movement significantly in either group (p>0.05). Similar to previous experiment, increasing the acceleration to 0.05g increased the rate of tooth movement over the OTM group in both 10 cN and 25 cN groups (2.4-fold) which in comparison with OTM group and 0.01g group was statistically significant (p<0.05). Increasing the acceleration to 0.1g increased the rate of tooth movement in comparison with OTM group 2.7- to 2.8-fold in the 10 cN and 25c N groups, respectively, which were statistically significant (p<0.05). Even though, a higher magnitude of tooth movement was observed in response to 0.1g HFA treatment compared to the response seen with 0.05g HFA treatment, the difference between the two groups was not significant (p>0.05). Since no significant difference was observed between 10 cN and 25 cN force in response to different acceleration, only data on 10cN orthodontic force are presented in all the remaining experiments.

To examine if increased acceleration is accompanied by increased loading of the alveolar bone, the average peak strain of different accelerations was calculated at the level of the buccal alveolar bone of the maxillary first molar. No significant differences in the magnitude of the average peak strain between static load and 0.01g were observed (p>0.05) (Fig 1E). However, increasing acceleration to 0.05g and 0.1g was accompanied by an increase in strain in the alveolar bone to 27 με and 55 με, respectively (Fig 1E). These changes were statistically significant when compared to the static load and 0.01g acceleration (p<0.05).

Since application of 0.1g may be uncomfortable for the animals (due to greater force on the tooth and higher peak strain in the alveolar bone), all remaining experiments were performed using 0.05g acceleration.

To test if the load produced in response to acceleration is the cause of the increase in the rate of tooth movement, the effect of similar peak strains under dynamic and static conditions were compared. Application of 30με static load (registered at the buccal plate of alveolar bone adjacent to first molar) increased the rate of tooth movement 1.3-fold, which was not significant compared with the OTM group (p>0.05) (Fig 1F). However, the dynamic load (HFA 120 Hz) that produced a similar load of 30με increased the rate of tooth movement more than 2-fold (p<0.05), suggesting load by itself was not the critical variable; instead, the nature of applied load (i.e. dynamic rather than static) was very important.

### Effect of frequency and time on rat of tooth movement is not linear

**Fig 2:**
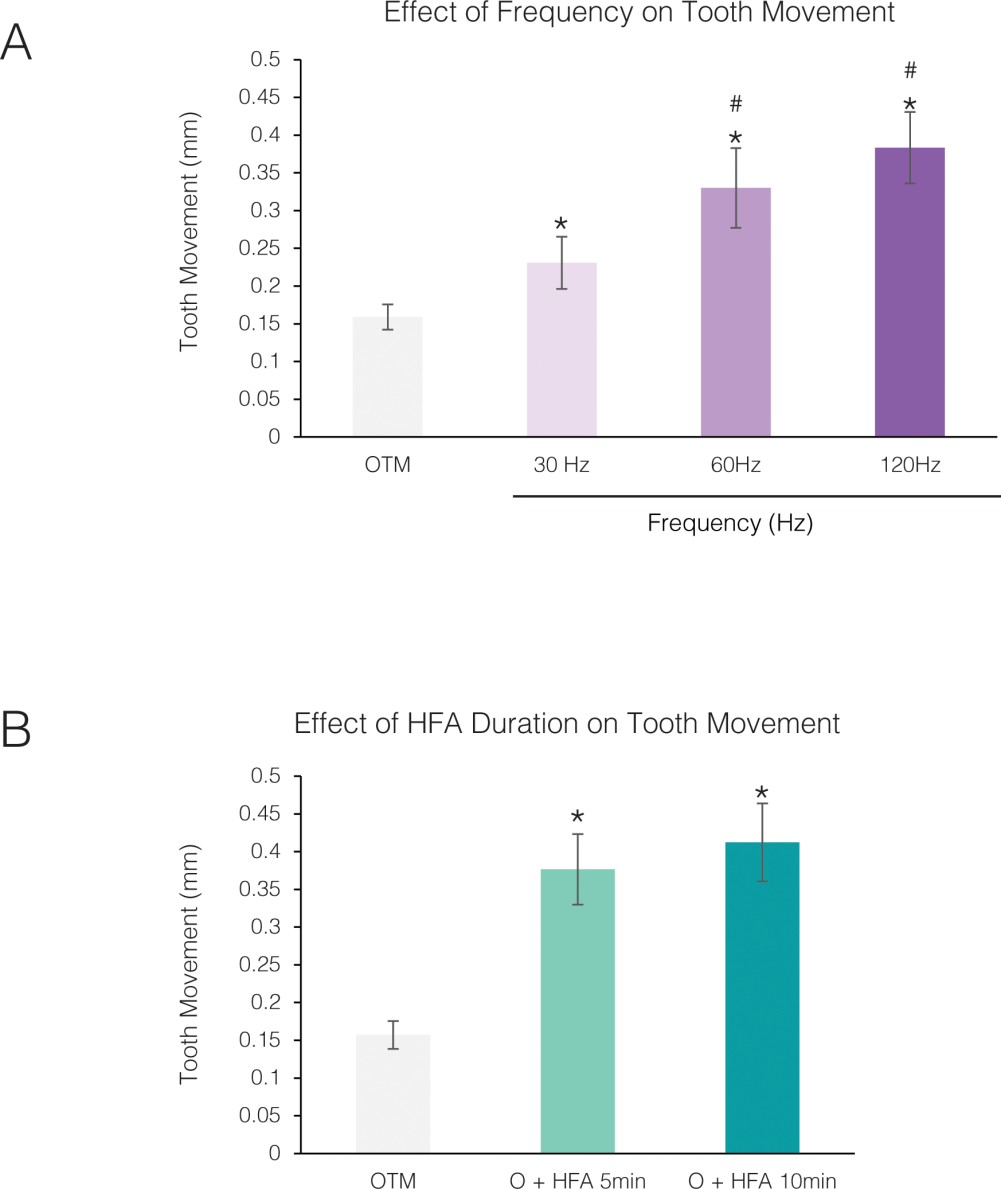
Effect of frequency and duration of HFA on the rate of tooth movement is not linear. **(A)** Effect of different frequencies on the rate of tooth movement under 0.05 g acceleration, 5 minutes per day for 14 days, in the presence of 10 cN orthodontic force (* Significantly different from OTM; ^#^ significantly different from 30Hz). (**B**) Effect of duration of HFA application on the rate of tooth movement under 120 Hz, 0.05g acceleration for 14 days in the presence of 10cN orthodontic force. Each value represents the mean ± SEM of 4 samples (* Significantly different from OTM).

To investigate the effect of frequency on the rate of tooth movement, different frequencies were applied to the maxillary right first molar, keeping all other parameters set at 0.05 g acceleration, 5 minutes per day for 14 days (Fig 2A). Compared to the OTM group, animals exposed to 30Hz demonstrate 1.45-fold increase in the rate of tooth movement. This increases in the rate of tooth movement in comparison with OTM group was statistically significant (p<0.05). Furthermore, increasing the frequency to 60 Hz and 120 Hz caused a 2.1- and 2.4-fold increase in the rate of tooth movement, respectively, that were statistically significant (p<0.05) compared with both OTM and 30Hz. While 120Hz demonstrate better results than 60 Hz, this difference was not statistically significant (p>0.05).

To evaluate if the duration of HFA application is an important factor in tooth movement, animals were exposed to HFA (120 Hz, 0.05 g) for 5 or 10 minutes per day for 14 days. Increasing the exposure to HFA increased the rate of tooth movement from 2.4-fold to 2.6-fold, which was not statistically significant (p>0.05) (Fig 2B). Based on these results, HFA stimulation parameters for the remaining experiments were set at 120 Hz, 0.05 g, and 5 minutes per day.

### Effects of HFA on the rate of tooth movement are similar to orthodontic forces: Both are PDL-dependent and enhance cytokine release

**Fig 3:**
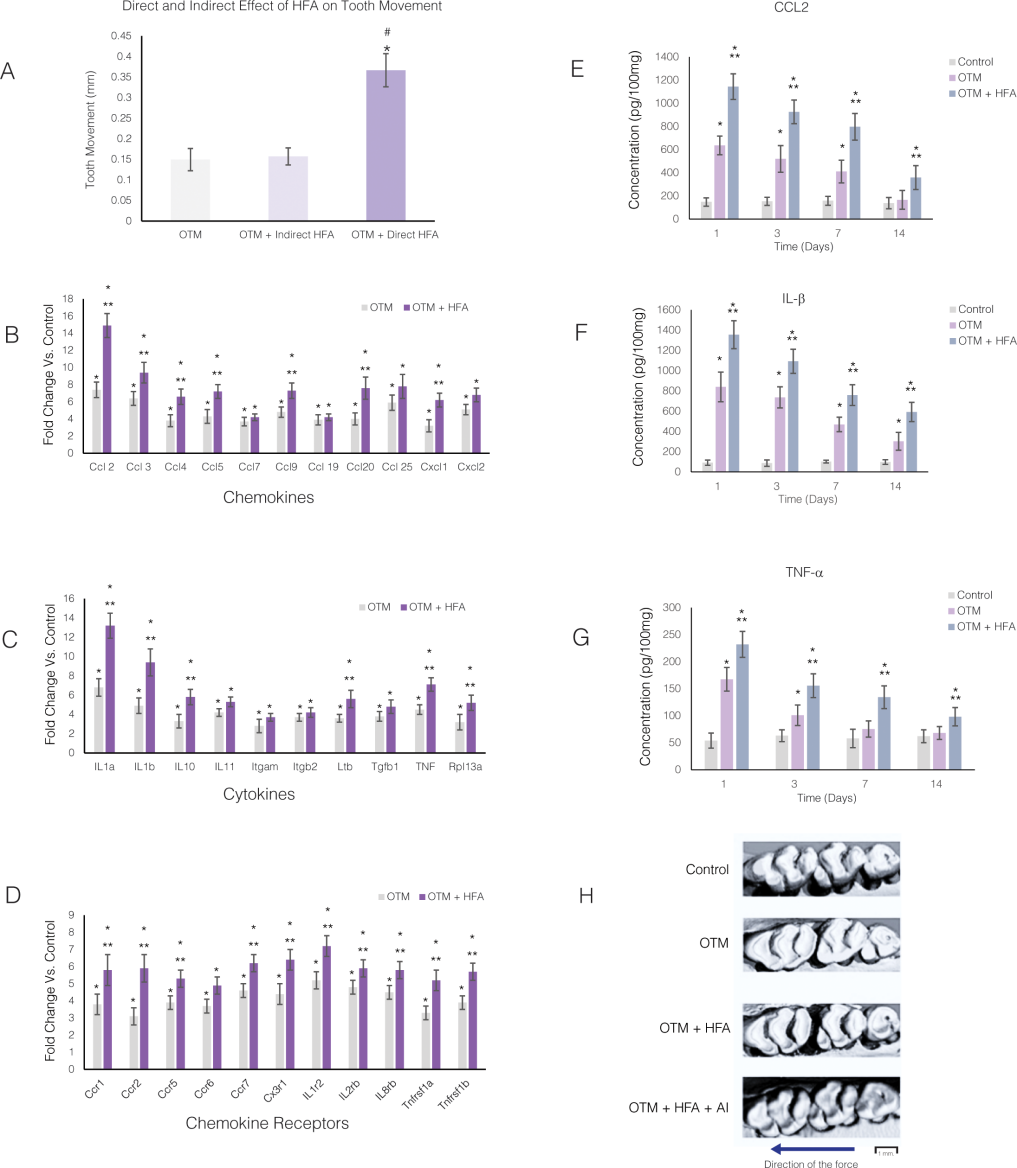
HFA effect on orthodontic tooth movement is PDL-dependent and through enhancement of cytokine release. **(A)** Animals were exposed to orthodontic force (OTM) alone or in combination with direct application of HFA for 5 minutes per day on the occlusal surface of the maxillary right first molar or indirect application of HFA on the adjacent tooth. HFA was calibrated so that the accelerometer measured the same acceleration and frequency on the buccal cortical plate of the maxillary first molar (120 Hz, 0.05g) in response to both direct or indirect loading. Tooth movement was measured in occlusal μCT images as the distance between the maxillary right first and second molars after 14 days. Each value represents the mean ± SEM of 4 samples. (* Significantly different from OTM group; ^#^ significantly different from Indirect load group). Mean “fold” increase in mRNA levels of different chemokines **(B)**, cytokines **(C)**, and their receptors **(D)** in maxillary right alveolar bone 24 hours after application of orthodontic force in the presence or absence of HFA (120 Hz, 0.05g) mechanical stimulation. Data expressed as the mean ± SEM of 5 samples (* Significantly different from control; ** significantly different from OTM). Mean protein concentration of CCL2 **(E)**, IL-1ß **(F)**, and TNF-*α* **(G)** in the maxillary right alveolar bone after 1, 3, 7 and 14 days of OTM or OTM + HFA. Data expressed as the mean ± SEM of concentration in picograms per 100 mg of tissue of 5 samples (* Significantly different from control; ** significantly different from OTM group). (**H**) Representative occlusal view of microCT images of right maxillary molars 14 days after application of orthodontic forces in OTM and OTM+HFA groups in absence or presence daily anti-inflammatory medication (OTM+HFA+AI). These images demonstrate blockage of stimulatory effect of HFA on rate of orthodontic tooth movement by anti-inflammatory medication. Arrow demonstrate the direction of the force application.

To determine if the effect of HFA on alveolar bone resulting in increased rate of tooth movement is mediated by the PDL, HFA was applied either directly to the maxillary first molar (the tooth being moved) or indirectly to the adjacent teeth (non-moving teeth) to bypass the influence of the first molar PDL. Experiments were calibrated so that the accelerometer registered similar frequency and acceleration in the buccal plate of the alveolar bone surrounding the maxillary first molar, in both scenarios. No difference in the rate of tooth movement was observed between the OTM group and the group that received indirect HFA (p>0.05) (Fig 3A). Only HFA applied directly to the first molar significantly increased the rate of tooth movement (p<0.05), which supports the importance of PDL in the HFA effect.

Since the PDL plays an important role in the HFA-induced increase in the rate of tooth movement, and because we have previously shown that the PDL is necessary for orthodontic tooth movement [2], we evaluated the expression of 32 cytokines, chemokines, and their receptors, that increase in the PDL in response to orthodontic forces [2]. Orthodontic forces were applied for 24 hours, in the absence or presence of HFA stimulation (120 Hz, 0.05g, 5 min). In addition, the effect of HFA on the expression of inflammatory markers was studied in the absence of orthodontic forces.

Compared to the Control group (received spring but no activation), the expression of 32 chemokines (Fig 3B), cytokines (Fig 3C), and their receptors (Fig 3D) increased more than 2-fold in both the OTM and OTM+ HFA groups. The range of expression was 2.8- to 7.4-fold in the OTM group and 3.7- to 14.2- fold in OTM + HFA group. All changes were statistically significant when compared to the Control group (p<0.05). All genes expressed in the OTM group were also expressed in the OTM + HFA group. Of the 32 genes, 24 were expressed significantly higher in OTM + HFA group compared to the OTM group (p<0.05). Expression of cytokines in the contralateral hemi-maxillae of both the OTM and OTM + HFA groups showed no statistically significant differences from the Control group (S1 Fig). In addition, no difference between expression of inflammatory markers in the HFA only group (no orthodontic force) and Control group was observed after 24 hours (S1 Fig) (p>0.05), which argues that orthodontically-induced inflammation is necessary for HFA to increase the rate of tooth movement. As previously reported [2] no difference between baseline animals (no spring installed or HFA treatment) and control group (spring installed without activation) was observed (S1 Fig).

To study the effect of HFA on inflammatory markers over a longer time period, protein levels of selected chemokines and cytokines were measured by ELISA at Days 1, 3, 7 and 14 after force application. The concentration of CCL2 (Fig 3E) increased significantly in the OTM group at Days 1, 3, and 7, which was statistically significant when compared to the Control group (p<0.05). No differences in CCL2 concentration were observed between the Control and OTM groups at Day 14 (p>0.05). However, the concentration of CCL2 at all time points studied was significantly higher in the OTM + HFA group when compared to both the Control and OTM groups (p<0.05).

The concentration of IL-1ß was significantly higher in both the OTM and OTM + HFA groups at all time points when compared to the Control group (p< 0.05) (Fig 3F). This increase was even higher in the OTM + HFA group compared to the OTM group for all time points (p<0.05).

TNF-*α* concentration was higher at Days 1, 3 and 7 in both the OTM and OTM+ HFA groups (p>0.05) (Fig 3G). At Day 14, no difference was observed in OTM group compared with control, while the OTM + HFA group still showed higher levels of TNF-*α* that was statistically significant (p<0.05).

To verify that elevated inflammatory mediators are required for HFA-enhanced orthodontic tooth movement, animals that received orthodontic forces and HFA were given anti-inflammatory (AI) medication (NSAIDs) for 14 days. Without NSAIDs, HFA enhanced orthodontic tooth movement compared to OTM, while NSAID treatment blocked HFA-enhanced orthodontic tooth movement (Fig 3H).

### HFA increased osteoclast activity and bone resorption during orthodontic tooth movement

**Fig 4:**
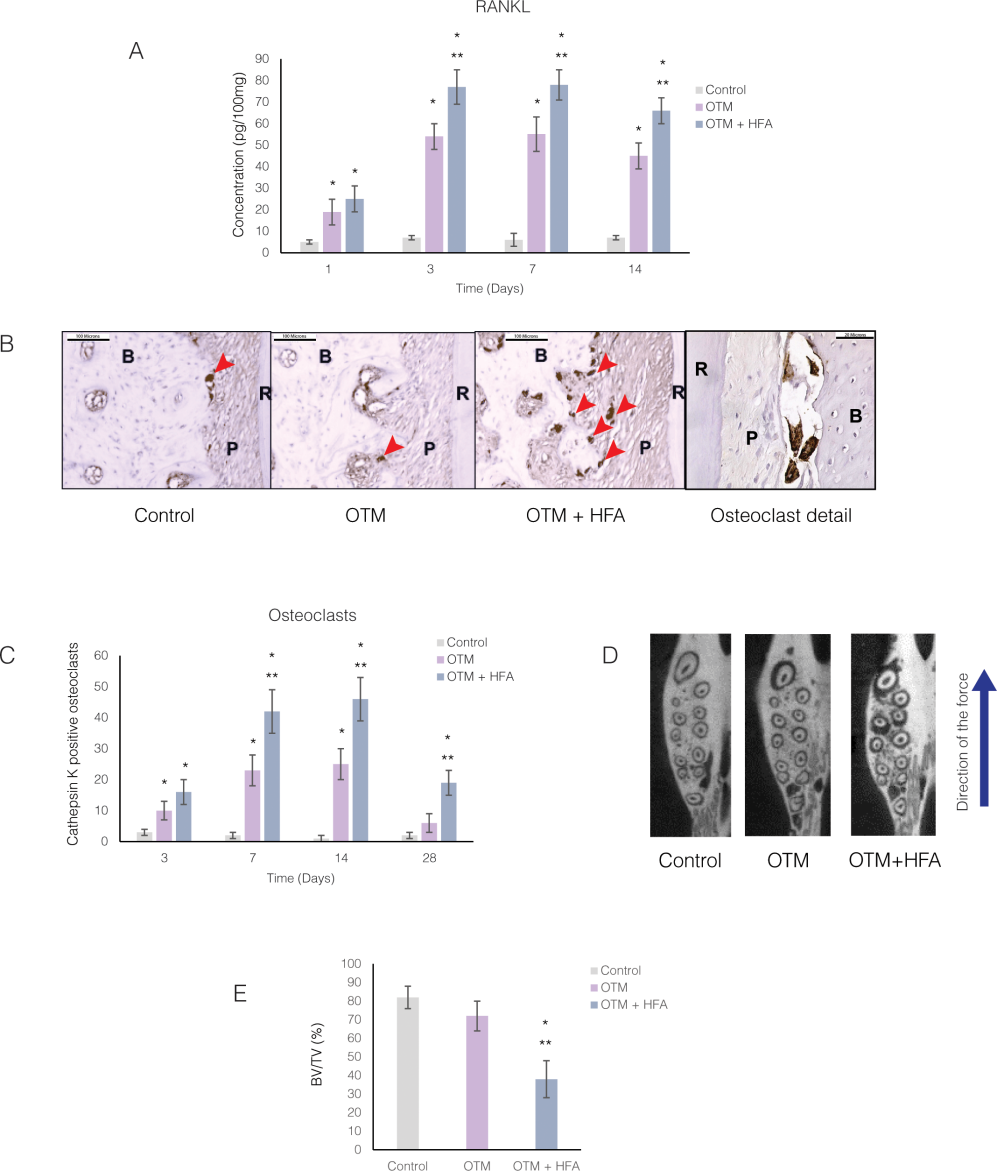
Osteoclast markers and number of osteoclasts increase in response to HFA mechanical stimulation. **(A)** Mean concentration of RANKL protein in the maxillary right alveolar bone after 1, 3, 7 and 14 days as measured by ELISA. Data expressed as the mean ± SEM of RANKL concentration in picograms per 100 mg tissue of 5 samples. (* Significantly different from control; **significantly different from OTM group). **(B)** Light microphotographs of Cathepsin K–positive osteoclasts in immunohistochemically stained sections of mesiopalatal root of the maxillary first molar. Images were collected close to the alveolar crest 28 days after application of force. Osteoclasts are stained as brown multinucleated cells *(arrow heads*) in sections from different groups and in the detail higher magnification. **(C)** Mean number of osteoclasts at different time points, in PDL and adjacent alveolar bone of mesiopalatal root of maxillary first molar. Each value represents the mean ± SEM of four animals (*Significantly different from control; **significantly different from OTM group). **(D)** Representative axial μCT sections showing decrease in bone density in OTM+ HFA at 14 days. Arrow demonstrates the direction of force application. **(E)** Average bone volume fraction (bone volume/total volume [BV/TV]) was calculated for Control, OTM and OTM+HFA group for the alveolar bone in the area of the maxillary first molar. Each value represents the mean ± SEM of four animals. (* Significantly different from control; ** significantly different from OTM group).

Having demonstrated that HFA increased the rate of orthodontic tooth movement and that this response required inflammatory mediators, we next investigated whether osteoclast recruitment to the site of HFA application (the maxillary first molar) increases with HFA treatment. We first performed ELISA for the osteoclast differentiation factor RANKL in rats treated with HFA for 1, 3, 7, and 14 days (Fig 4A). RANKL concentration in the OTM group significantly increased at each time point (3.7- to 9.1- fold) compared with the Control group, with the peak at Day 7 (p<0.05). The OTM + HFA group also showed a significantly higher concentration of RANKL compared to the Control group (5- to 13- fold) with a peak at Day 7. The increase in RANKL concentration in the OTM + HFA group was statistically significant for all time points studied in comparison to both Control and OTM groups (p<0.05).

To investigate if the increase in the osteoclast differentiation factor RANKL leads to increased osteoclastogenesis, we counted Cathepsin K positive (CK+) cells in immunohistochemically stained sections of the root and adjacent alveolar bone of the maxillary first molar (Fig 4B). Cathepsin K is a marker of mature osteoclasts. The number of CK+ multinucleated cells significantly increased in both the OTM and OTM + HFA groups at all time points, with a peak increase at Day 14 (Fig 4C). The OTM + HFA group showed a statistically significant increase in the number of CK+ cells compared to the OTM group for all time points (p<0.05). At Day 28, while the number of CK+ cells in the OTM groups decreased significantly, the HFA group still presented a statistically significant increase in CK+ cells. Cathepsin K expression was strongest in high-stress areas (adjacent to alveolar crest in the direction of tooth movement) and at the root apex area opposite to the direction of tooth movement. The increase in osteoclast number was observed mostly on alveolar bone surface adjacent to the PDL side and not on the endosteal side. Also, osteoclasts were not limited to the compression side and were found in both the compression and tension sides.

With an increase in osteoclasts around maxillary first molars receiving HFA, we next used microCT quantification to evaluate the effects of HFA on alveolar bone density during tooth movement (Fig 4D). Bone volume fraction (BV/TM) levels in the OTM + HFA group was significantly lower (p<0.05) than in the Control or OTM groups (Fig 4E). After 14 days of treatment, the mean BV/TV fraction around the maxillary first molar in the Control (82%) and OTM (73%) groups were not statistically different (p>0.05). However, the mean BV/TV in the OTM + HFA group decreased to 44%, which is significantly less (p<0.05) compared to both the Control and OTM groups. This effect was limited to the left hemi-maxillae, and no change in BV/TV fractions was observed in contralateral hemi-maxillae (p>0.05) (S2 Fig).

## Discussion

To our knowledge, our experiments demonstrate for the first time that vibration in the form of HFA produces a significant localized catabolic effect strong enough to increase the rate of tooth movement in response to orthodontic forces. Our findings create an interesting paradox, since orthodontic tooth movement depends on osteoclast activation and bone resorption, while under physiological conditions, HFA produces a significant anabolic effect on the craniofacial skeleton (Table 1). To better understand HFA’s paradoxical effects on alveolar bone, we analyzed the biological phenomena underlying the novel catabolic effect of HFA during orthodontic tooth movement.

**Table 1:** Summary and distinguishing characteristics of the anabolic and catabolic effect of HFA and their possible clinical applications in orthodontics treatment.

	Anabolic effect	Catabolic effect
**Method of application**	• Directly on teeth in the target area, or • Indirectly on adjacent teeth close to the target area	• Directly on tooth or teeth that are moving
**Initial state of tissue**	• Physiologic condition	• Inflammatory condition
**Target tissue**	• Bone	• Periodontal ligament
**Responding cells**	• Osteocytes • Osteoblasts	• Osteoclasts
**Resulting effect**	• Bone formation • Load-independent	• Bone resorption • Load-dependent
**Extension of effect**	• Gradient effect with highest response on bone surrounding target tooth and extending to adjacent bone	• No gradient effect, effective only on target tooth exposed to orthodontic forces with no effect on adjacent teeth
**Potential clinical uses**	• Preservation of alveolar bone after extractions • Bone regeneration after periodontal disease • Enhance Implant and graft integration • Increased bone formation after Orthopedic treatment • Improved retention after Orthodontic treatment • Increased bone formation after Orthognathic Surgery	• Accelerated tooth movement • Increase in magnitude of movement (distance) • Differential anchorage • Increase in magnitude of Orthopedic correction • Reduced bone density around target tooth to facilitate different types of tooth movement • Reduced necrotic (hyalinized) area in response to static Orthodontic forces • Possible increased frontal resorption

One major contributor to HFA’s unexpected catabolic effect in our studies is the baseline condition of the tissues exposed to HFA stimulation. Anabolic effects of HFA are consistently observed in tissues that are healthy or in a repair phase [10–14]. What these conditions have in common are low levels of inflammatory mediators. In our studies, orthodontic forces imposed an insult on the PDL that initiated and sustained the release of inflammatory mediators [27, 28] into the area for as long as orthodontic force was applied. Therefore, it is logical to conclude that in the presence of elevated levels of inflammatory mediators, HFA intensifies the existing inflammation, while under physiological conditions HFA does not stimulate inflammation. This conclusion is supported by our experiments that show HFA induced expression of inflammatory markers only in the presence of orthodontic forces. Without orthodontic forces–and the inflammation that accompanies them–no HFA-induced increase in inflammatory markers was observed. This is in agreement with a recently published article that demonstrates vibration increases the rate of tooth movement only in the presence of continuous orthodontic forces [29].

The inflammatory markers are not only necessary for orthodontic tooth movement, they are the main mechanism through which HFA increased the rate of tooth movement, since treatment with NSAIDs decreased the rate of HFA-induced tooth movement. This is consistent with published data demonstrating the inhibitory effect of NSAIDs on orthodontic tooth movement [30–32].

While we show that HFA increased inflammatory marker expression, it was not clear if the source of the markers was alveolar bone or the PDL. To address this question, we first examined the effect of direct and indirect HFA on the rate of tooth movement. Direct HFA treatment on the moving tooth was applied directly to the crown, thus the direct HFA stimulation vector went from the crown, through the PDL to the alveolar bone. Conversely, indirect HFA was applied to the crown of the adjacent tooth, thus the indirect HFA stimulation vector went from the crown of the non-moving tooth, though its PDL into the alveolar bone around the moving tooth. If the indirect HFA stimulation was detected by the moving tooth’s PDL, it could be argued that the indirect HFA stimulation would be greatly attenuated compared to the intensity felt through direct HFA stimulation, thus accounting for the lack of a catabolic response following indirect HFA stimulation. To ensure that this would not be the case, we equilibrated the frequency, acceleration and strain in the buccal plate of the alveolar bone surrounding the maxillary first molar in both experimental set ups. Our study showed that only direct application of HFA (to the moving tooth) increased the rate of orthodontic tooth movement. Indirect application of HFA (to the adjacent tooth) did not intensify the catabolic effect of orthodontic forces. From these data we conclude that the direct disturbance of PDL already under the trauma of orthodontic forces is necessary for HFA’s catabolic effect to occur. This observation is in agreement with previous studies that have shown that the source of inflammatory markers during orthodontic tooth movement is the PDL [1–4] and clinical observations that in the absence of a PDL (for example, ankylosed tooth or implant) there is no orthodontic tooth movement.

The observation that catabolic effects of HFA are similar to orthodontic forces in that both target the PDL, is strikingly different from HFA’s anabolic effect in which bone formation occurs in response to both direct and indirect application of mechanical stimulation in absence of any inflammatory inducing factor, such as orthodontic forces [10, 11]. Therefore, together these studies support our conclusion that while the direct target of the HFA anabolic effect is bone, the direct target of the HFA catabolic effect is the PDL (Table 1). This conclusion is supported by a previous study [33] showing that a 60Hz, 1m/s^2^ vibration applied directly to the maxillary first molars undergoing expansion demonstrate significantly increased rate of tooth movement, RANKL expression in PDL cells, and PDL osteoclast number.

It could be argued that the source of inflammatory markers is alveolar bone and not the PDL. HFA may induce micro-fractures in bone, which initiates bone remodeling and thereby accelerates tooth movement [34–37]. Three facts argue against this proposal. First, HFA in the absence of orthodontic forces is anabolic, thus negating the requirement for micro-fractures to have the catabolic effect. Second, indirect application of HFA—with or without orthodontic force—does not accelerate the rate of tooth movement (even though bone would be equally likely to sustain micro-fractures). Third, the majority of osteoclasts were located on the PDL side, rather than on the endosteal side, of the alveolar bone. if micro-fractures were the source of osteoclasts, one would expect the number of osteoclasts to increase on both the PDL and endosteal sides of the alveolar bone. Taken together with published works, our data support the conclusion that the PDL is the target and mediator of HFA’s catabolic effects.

Another phenomenon that needs an explanation is why HFA increased the already high level of inflammatory markers. Since expression of inflammatory markers in response to orthodontic forces depends on the magnitude of the applied force, it is logical to assume the effect of HFA on expression of inflammatory markers in the PDL may also depend on the magnitude of the load applied to the tooth. We have previously shown that there is a saturation point above which the application of higher magnitudes of force does not increase the expression of inflammatory markers. Therefore, osteoclast recruitment, and the resulting rate of tooth movement plateau [12]. For any procedure to accelerate the rate of tooth movement beyond this biological limit, it must raise the saturation point. In the current experiments, we applied both 10 cN and 25cN orthodontic forces, a force we showed is above the level of the biological saturation point force in rats [9]. As in the previous study, we found no significant difference in the magnitude of tooth movement between 10cN and 25 cN forces. The fact that increasing the static force did not increase the rate of tooth movement argues that the magnitude of the load produced by vibration alone cannot explain the increase in the rate of tooth movement. Our finding that applying HFA for a short period of time (5 min per day) increased the expression of inflammatory markers beyond the saturation point, while simultaneously increasing the rate of tooth movement, suggests that HFA not only bypasses the saturation point limitation of orthodontic forces, but supports a novel reaction of the PDL and bone remodeling system to the vibration in the presence of inflammation. Indeed, there is clear evidence that the biological status of bone can affect the magnitude of response of bone to vibration [38]. How exactly these responses are mediated is unclear and, like our data here, point to the need for in-depth investigations into the molecular and cellular responses of bone to vibration.

Our studies demonstrated that different characteristics of vibration can significantly affect the rate of tooth movement. In this regard acceleration play a significant role in rate of tooth movement. However, from a practical point of view, there is a limit to how much we can increase acceleration, since an increase in acceleration is accompanied by an increase in loading of the PDL and alveolar bone that can be uncomfortable. Under clinically acceptable acceleration, increasing frequency can increase the rate of tooth movement. However, after a certain level of frequency and duration, the effect plateaus and no further increase in the rate of tooth movement is observed. This observation demonstrates that similar to constant orthodontic forces, dynamic stimulation may also reach a biological saturation point, which requires further clarification. Therefore, based on these results, the physical characteristics of vibration should be more clearly defined before we can compare the results of different studies. For example, some of the previous animal studies concluding that vibration cannot increase the rate of tooth movement, have used lower acceleration and lower frequencies [16, 19]. Our current study confirmed that lower acceleration and lower frequency are not very effective in increasing the rate tooth movement.

One of the main concerns about using HFA to increase the rate of tooth movement is the possibility of increasing root resorption due to prolonged exposure of roots to inflammatory mediators. While we did not study root resorption in our study, others have shown no indication of root resorption in response to vibration [29, 39]. Moreover, we found that while HFA increased the number of osteoclasts, all osteoclasts were located on the alveolar bone surface, rather than root surface, which argues against the idea that HFA may contribute to root resorption. Prolonged expression of inflammatory markers in response to HFA may actually prevent root resorption by decreasing the bone density through which roots are moved. Further research in this area is required.

Our study suggests for first time that HFA treatment during orthodontic tooth movement produces a catabolic effect that has a significant clinical impact. These results, together with our previous study that showed a striking anabolic effect of HFA on alveolar bone when applied to healthy teeth [10, 11], strongly supports a revolutionary role for this safe, non-invasive technique in orthodontics. Not only can the catabolic effect of HFA accelerate the rate of orthodontic and orthopedic treatment, but in the absence of orthodontic forces, HFA can induce an anabolic response with the potential to increase the stability and retention of orthodontic results.

## Supporting information

S1 Fig Cytokine levels under different experimental conditions. Mean “fold” increase in mRNA levels of different cytokines, CCL2 (A), IL-1 ß (B) and TNF-*α* (C) in maxillary right alveolar bone 24 hours after application of orthodontic force in the presence or absence of HFA (120 Hz, 0.05g) mechanical stimulation. Data expressed as the mean ± SEM of 5 samples (*significantly different from Baseline; ** significantly different from OTM). Baseline (group that did not receive any spring or mechanical stimulation), HFA (group that received only HFA in absence of any spring), OTM (Orthodontic tooth movement group, that received active spring), OTM+ HFA (Orthodontic tooth movement group that received active spring and HFA treatment), CL-OTM (Contra-lateral side of OTM group, that did not receive active spring), CL-OTM+HFA (Contra-lateral side of OTM+HFA group, that did not receive active spring or HFA).
(PDF)

S2 Fig Bone volume quantification by μCT analysis for different experimental conditions. Average alveolar bone volume fraction (bone volume/total volume [BV/TV]) was calculated in the area of the maxillary first molar for Control, OTM (Orthodontic tooth movement group, that received active spring), contra-lateral side of OTM group (CL-OTM, that did not receive active spring), OTM+ HFA (Orthodontic tooth movement group that received active spring and HFA treatment), contra-lateral side of OTM-HFA group (CL-OTM + HFA, that did not receive active spring or HFA). Each value represents the mean ± SEM of four animals. (*significantly different from control; ** significantly different from OTM group).
(PDF)
