# Supplementary material for: Vibration paradox in orthodontics: Anabolic and catabolic effects
Source: PLoS One. 2018 May 7;13(5):e0196540. doi: 10.1371/journal.pone.0196540 (PMC5937741; doi:10.1371/journal.pone.0196540)
Supplement: S1 Fig — Mean “fold” increase in mRNA levels of different cytokines, CCL2 (A), IL-1 ß (B) and TNF-α (C) in maxillary right alveolar bone 24 hours after application of orthodontic force in the presence or absence of HFA (120 Hz, 0.05g) mechanical stimulation. Data expressed as the mean ± SEM of 5 samples (*significantly different from Baseline; ** significantly different from OTM). Baseline (group that did not receive any spring or mechanical stimulation), HFA (group that received only HFA in absence of any spring), OTM (Orthodontic tooth movement group, that received active spring), OTM+ HFA (Orthodontic tooth movement group that received active spring and HFA treatment), CL-OTM (Contra-lateral side of OTM group, that did not receive active spring), CL-OTM+HFA (Contra-lateral side of OTM+HFA group, that did not receive active spring or HFA). (PDF) [file pone.0196540.s001.pdf]

S1 Figure

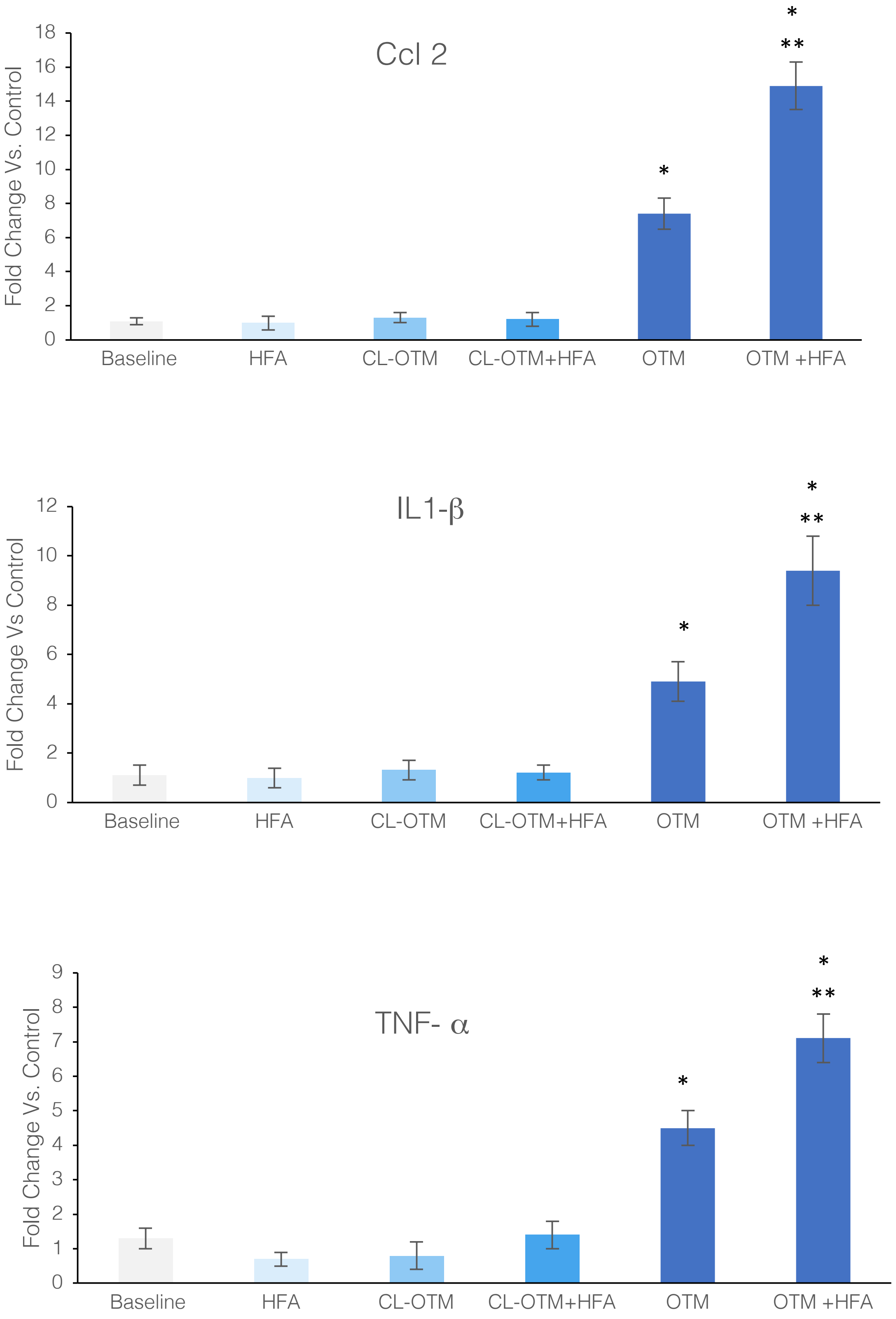

Ccl 2
Fold Change Vs. Control
18
16
14
12
10
8
6
4
2
0
Baseline
HFA
CL-OTM
CL-OTM+HFA
OTM
OTM +HFA
*
**
IL1-β
Fold Change Vs Control
12
10
8
6
4
2
0
TNF- α
Fold Change Vs. Control
9
8
7
6
5
4
3
2
1
0

S1 Figure Legend:

Cytokine levels under different experimental conditions. Mean “fold” increase in mRNA levels of different cytokines, CCL2 (A) , IL-1β (B) and TNF-α (C) in maxillary right alveolar bone 24 hours after application of orthodontic force in the presence or absence of HFA (120 Hz, 0.05g) mechanical stimulation. Data expressed as the mean ± SEM of 5 samples (* significantly different from Baseline; ** significantly different from OTM). Baseline (group that did not receive any spring or mechanical stimulation), HFA (group that received only HFA in absence of any spring), OTM (Orthodontic tooth movement group, that received active spring), OTM+ HFA (Orthodontic tooth movement group that received active spring and HFA treatment), CL-OTM (Contra-lateral side of OTM group, that did not receive active spring), CL-OTM+HFA (Contra-lateral side of OTM+HFA group, that did not receive active spring or HFA).
