# Supplementary material for: Vibration paradox in orthodontics: Anabolic and catabolic effects
Source: PLoS One. 2018 May 7;13(5):e0196540. doi: 10.1371/journal.pone.0196540 (PMC5937741; doi:10.1371/journal.pone.0196540)
Supplement: S2 Fig — Average alveolar bone volume fraction (bone volume/total volume [BV/TV]) was calculated in the area of the maxillary first molar for Control, OTM (Orthodontic tooth movement group, that received active spring), contra-lateral side of OTM group (CL-OTM, that did not receive active spring), OTM+ HFA (Orthodontic tooth movement group that received active spring and HFA treatment), contra-lateral side of OTM-HFA group (CL-OTM + HFA, that did not receive active spring or HFA). Each value represents the mean ± SEM of four animals. (*significantly different from control; ** significantly different from OTM group). (PDF) [file pone.0196540.s002.pdf]

S2 Figure

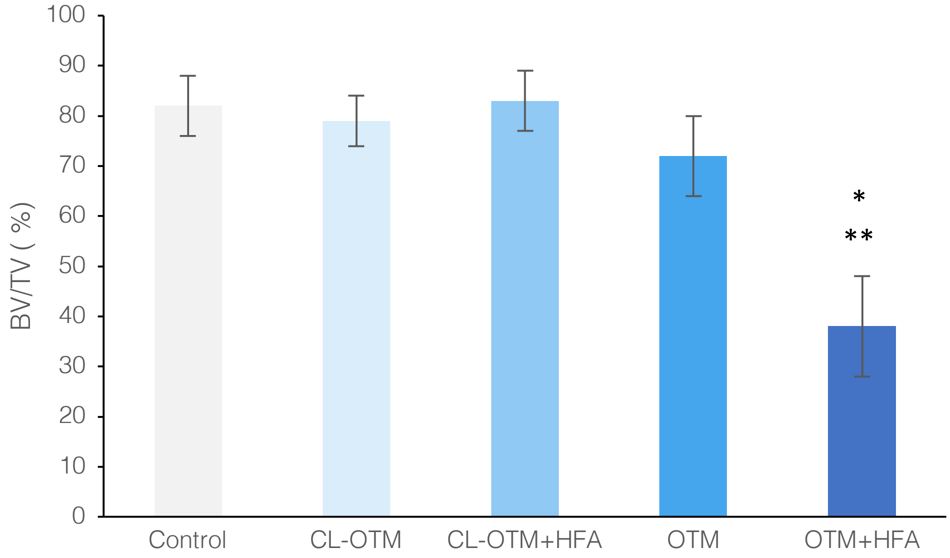

S1 Figure Legend:

Bone volume quantification by uCT analysis for different experimental conditions. Average alveolar bone volume fraction (bone volume/total volume [BV/TV]) was calculated in the area of the maxillary first molar for Control, OTM (Orthodontic tooth movement group, that received active spring), contra-lateral side of OTM group (CL-OTM, that did not receive active spring), OTM+ HFA (Orthodontic tooth movement group that received active spring and HFA treatment), contra-lateral side of OTM-HFA group (CL-OTM + HFA, that did not receive active spring or HFA). Each value represents the mean ± SEM of four animals. (* significantly different from control; ** significantly different from OTM group).
